# Sustained High‐Loneliness States in Persistent Depressive Disorder: An Ecological Momentary Assessment Study

**DOI:** 10.1111/nyas.70436

**Published:** 2026-10-23

**Authors:** Johannes Wolf, Katrin Wörz, Amelie Cronau, Franziska Midderhoff, Julia I. Kunz, Alma‐Sophia Merscher, Claus Vögele, Andrea Jobst, Stephan Goerigk, Frank Padberg, Matthias A. Reinhard

**Affiliations:** ^1^ Department of Psychiatry and Psychotherapy LMU University Hospital, LMU Munich Munich Germany; ^2^ German Center for Mental Health (DZPG) Partner Site Munich‐Augsburg Munich Germany; ^3^ Department of Psychology Ludwig‐Maximilians‐University Munich Munich Germany; ^4^ Department of Behavioural and Cognitive Sciences University of Luxembourg Esch‐sur‐Alzette Luxembourg; ^5^ Charlotte Fresenius Hochschule Munich Germany

**Keywords:** early adversity, ecological momentary assessment, emotional isolation, persistent depressive disorder, state loneliness

## Abstract

Loneliness is a risk factor for mental disorders. While transient loneliness states are thought to serve as an adaptive signal motivating social reconnection, elevated loneliness states that persist may turn maladaptive. Yet, how the level and persistence of loneliness states differ among individuals with and without mental disorders remains unclear. We used ecological momentary assessment (EMA) to characterize state loneliness in inpatients with persistent depressive disorder (PDD; *n* = 58) and in healthy controls (HCs; *n* = 42), deriving two theoretically motivated indices: mean level and duration of high‐loneliness states. Trait loneliness (UCLA Loneliness Scale) and recalled early adversity (Childhood Trauma Questionnaire; emotional neglect and abuse) were also assessed. As hypothesized, inpatients with PDD reported higher mean state loneliness and longer high‐loneliness states, lasting 3.5 consecutive prompts on average. Inpatients with PDD were also less likely to leave a high‐loneliness state at the next assessment, adjusted for current level of loneliness. State emotional isolation (the momentary perception of lacking people to turn to) emerged as the most consistent correlate of trait loneliness and was associated with recalled emotional neglect. These exploratory findings suggest that early emotional adversity may contribute to emotional isolation in daily life and, in turn, to trait loneliness in PDD, requiring prospective testing.

## Graphical abstract

While transient loneliness may serve as an adaptive signal motivating social reconnection, persistent loneliness may become maladaptive. Ecological momentary assessment in 58 inpatients with persistent depressive disorder (PDD) and 42 healthy controls (HCs) revealed higher state loneliness and more persistent high‐loneliness states in PDD, including lower probability of leaving these states after adjusting for current level. State emotional isolation was associated with trait loneliness and recalled emotional neglect, motivating prospective investigation.

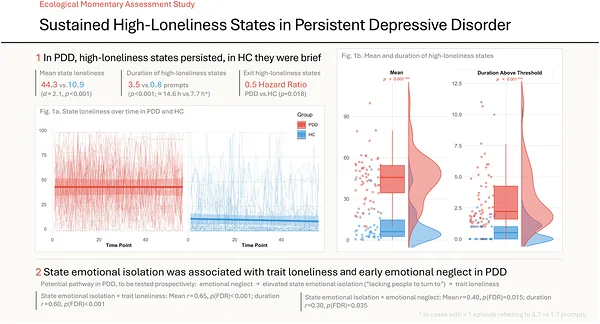

## 1 Introduction

Loneliness is a highly prevalent, aversive, and stigmatizing transdiagnostic condition in individuals with and without mental disorders [1]. In a European Union–wide survey conducted by the European Commission approximately 13% of participants reported feeling lonely most days in the last 4 weeks, while 35% reported loneliness at least some of the time [2]. Loneliness describes a distressing experience that arises when there is a discrepancy between desired and actual social relationships in terms of quantity and/or quality [3]. Therefore, loneliness can occur even in the presence of frequent social interactions [4, 5] and is experienced in different facets including a lack of close, confiding relationships (emotional isolation) and a more general lack of social contacts (social isolation) [3].

Loneliness constitutes a significant risk factor for mental disorders and for the worsening of symptoms of several mental and physical conditions such as depression, dementia, or cardiovascular conditions [6, 7, 8, 9, 10, 11, 12]. However, loneliness is not static: it ranges from transient states to persistently elevated levels and varies substantially within individuals over time [13, 14]. Transient loneliness states may serve an adaptive signaling function: as aversive experiences, they may indicate a lack of meaningful connection and motivate individuals to re‐establish social contact, thereby resolving themselves. When loneliness persists, this signal may turn maladaptive, fostering hypervigilance to social threat, negative social expectations, and withdrawal [13, 14, 15]. The level and persistence of loneliness states in daily life may thus be informative parameters regarding the adaptive or maladaptive signal. Yet loneliness has predominantly been measured as a stable trait via retrospective self‐report questionnaires, an approach that obscures these temporal characteristics of loneliness [14, 16, 17, 18, 19]. Ecological momentary assessment (EMA) addresses this limitation. Results from studies using EMA showed that state loneliness fluctuates considerably over time, is shaped by momentary social context and coping behavior [20, 21, 22], and is associated with the course of mental health challenges including depressive symptoms or suicidality [8, 14]. However, it remains largely unclear how the level and persistence of loneliness states differ between healthy and clinical populations [13, 14].

One clinical condition in which loneliness appears particularly relevant for symptom onset and course is persistent depressive disorder (PDD) [8, 23, 24, 25, 26]. Individuals with PDD reported higher levels of trait loneliness than both healthy individuals and those with episodic depression, and loneliness has been linked to higher symptom severity, poorer treatment outcomes, and increased suicidal ideation in this group [7, 8, 23, 27]. McCullough's model of persistent depression, the theoretical basis of the Cognitive Behavioral Analysis System of Psychotherapy (CBASP), offers an explanation for this close link: persistent depression, particularly with early onset, is proposed to arise from the experience of early adversity, including emotional neglect and abuse, which erode subjective interpersonal safety [28]. The resulting generalized fear of interpersonal closeness is managed through pervasive interpersonal avoidance, leaving patients perceptually disconnected from their social environment and unable to establish the connections that would meet their relational needs [28]. Empirical findings converge with this account: in both a population‐based and a clinical sample including PDD, trait loneliness was more closely tied to early adversity, here operationalized as childhood maltreatment, than the objective size of the social network was, and most strongly to emotional neglect and emotional abuse [23, 29]. Confirming this pattern, a meta‐analysis showed that individuals with early adversities feel substantially lonelier, with emotional neglect and abuse being more strongly associated with loneliness than other forms of adversity. This meta‐analysis explicitly called for the duration of loneliness states to be investigated, that is, whether loneliness is transient (a self‐limited state) or sustained (a prolonged state or even trait) [30]. Loneliness may thus represent one candidate pathway linking early adversity with PDD, although other causes and mechanisms are equally plausible [23, 28, 29, 30, 31].

Taken together, this framework suggests two measurable indices of state loneliness in daily life, which follow directly from the CBASP model as well as previous literature and may be informative regarding the adaptive versus maladaptive function of loneliness: unmet connection needs should manifest in elevated state loneliness (mean level); and, as proposed by McCullough's model, interpersonal avoidance in PDD may hinder the reconnection behavior through which transient loneliness normally resolves, resulting in the persistence of high‐loneliness states (duration of subsequent loneliness states above a certain threshold).

Against this background, the present study aimed to assess state loneliness in PDD compared to healthy controls (HCs). We hypothesized that the PDD group would exhibit elevated and more persistent loneliness states than HC. Based on the CBASP model and prior findings on early adversity [23, 29], we further explored how state loneliness would be associated with trait loneliness and early emotional neglect and abuse. As the hypothesis and analyses of this study were not preregistered, all findings should be considered exploratory and hypothesis‐generating.

## 2 Methods

### 2.1 Sample

The study investigated a clinical group of inpatients diagnosed with PDD, recruited using convenience sampling at the Department of Psychiatry and Psychotherapy, LMU University Hospital Munich. Participants received multimodal inpatient treatment during the assessment period. A comparison group of HC was recruited matching age, gender, and education, via social media postings, flyers, mailing lists, and online advertisement platforms. All participants underwent diagnostic assessment in accordance with DSM‐5 criteria using structured clinical interviews (SCID‐5‐CV and SCID‐5‐PD). Participants provided written informed consent prior to study participation. The study protocol was approved by the Ethics Committee of the Medical Faculty of LMU Munich (Reference number: 22‐0299). The registered recruitment target of the overarching study was 100 participants (study registration: DRKS00037153), originally planned as 50 per group. Because recruitment of HC proved more difficult while interest among the PDD group was high, the group ratio was adapted during recruitment while retaining the overall target. The present manuscript reports a secondary analysis of the full dataset available at the analysis cutoff date (April 1, 2026). One hundred and fourteen participants were enrolled by the cutoff date, of whom 100 (58 PDD, 42 HC) provided sufficient EMA data and constitute the present sample. In line with previous studies [32], participants with less than 50% answered prompts on the EMA loneliness variables across all time points were excluded from analyses (PDD: *n* = 7, HC: *n* = 7). The remaining participants showed an EMA adherence of 90.2% in PDD and 84.7% in HC. Given the continued high interest among inpatients with PDD, recruitment is ongoing beyond the registered target; these participants are not part of the present analysis. The decision to conduct these analyses was not contingent on any interim inspection of the data. The hypotheses and analysis pipeline of this secondary analysis were not preregistered.

### 2.2 Procedure

This was a prospective longitudinal study in a naturalistic setting over a period of 5 weeks. During the first and the fifth week, participants completed EMA questionnaires four times per day at randomized intervals between 8:00 a.m. and 8:00 p.m. with a minimum interprompt interval of 90 min. The median elapsed time between consecutive answered prompts was 204 min (interquartile range [IQR] 185 min), including overnight prompts, and 176 min (IQR 100 min) for within‐day pairs. Clinical assessments were administered at the beginning (first day of week 1) and end of the study period (last day of week 5).

### 2.3 Clinical Assessments

Trait loneliness was assessed using the UCLA‐LS [33, 34]. Three subscales assess subjective feelings of loneliness, perceived emotional and social isolation with a total of 20 items rated on a 5‐point scale from 1 (“not at all”) to 5 (“totally”). In addition, a total score was calculated. The German version of the UCLA‐LS showed high internal consistency (Cronbach's α = 0.89) [34]. Early adversity was assessed with the Childhood Trauma Questionnaire (CTQ) [35, 36], a measure of recalled child maltreatment. Analyses focused a priori on the emotional domain, that is, emotional neglect (Cronbach's α = 0.83) and emotional abuse (Cronbach's α = 0.87) [36], given previous evidence [23, 30]. For sample characterization, the severity of depressive symptoms was measured with the total score of the German version of the Beck Depression Inventory second version (BDI‐II) [37].

### 2.4 Ecological Momentary Assessment

EMA data were collected using the *movisensXS* application, which was installed on participants’ Android smartphones. EMA items were selected from established item pools used in previous studies [38]. In this study we used three loneliness items adapted from the subscales of the UCLA‐LS: state loneliness (“At the moment I feel lonely.”), state emotional isolation (“At the moment I have enough people I can turn to.”), and state social isolation (“At the moment I have enough company.”). The state prefix denotes the momentary perception of the respective facet; item labels follow the UCLA‐LS subscales from which the items were adapted. Participants rated EMA items on a visual analogue scale with poles “not at all” (0) and “very much” (100). The items for state emotional and social isolation were reversed for analyses. A detailed description of the EMA procedure and items can be found elsewhere [7].

### 2.5 Statistical Analyses

All analyses were conducted in R (version 4.5.1) using *tidyverse* for data processing and visualization, and *flextable* and *officer* for table generation. Figures were produced using *ggplot2* and *gridExtra*. Descriptive statistics were calculated for baseline demographic and clinical variables separately for PDD and HC. Two state loneliness indices were computed for each participant and for each of the three state loneliness items: (1) the mean across all available state values to measure elevation, and (2) duration above threshold to measure persistence. For the latter, a single global threshold was defined as the 75th percentile of EMA values pooled across all raw observations of both groups. Given high and similar adherence in both groups, thresholds were not participant weighted. A global rather than person‐specific threshold was chosen because the construct of interest was persistent, elevated loneliness states; person‐specific thresholds would, by construction, fail to identify persistent high‐loneliness states in individuals with chronically elevated loneliness. Numerical thresholds were 51 for state loneliness, 63 for state emotional isolation, and 55 for state social isolation, each on the 0–100 visual analogue scale (isolation items reverse‐coded). Runs were formed over the sequence of answered prompts and could continue from the last prompt of 1 day to the first prompt of the next; overnight intervals were not treated differently from within‐day intervals. Unanswered prompts were skipped and did not terminate a run; runs could not bridge the interval between assessment week 1 and week 5. Participants without any above‐threshold observation were assigned a value of 0 on the duration index. In addition, the elapsed duration of runs was computed from the prompt timestamps as the interval between the first and the last above‐threshold observation of a run, averaged within participants before averaging across participants. Group differences in the state loneliness indices were examined using independent‐samples *t*‐tests, with Cohen's *d* and its 95% confidence interval reported as effect sizes. Associations between the state indices and trait loneliness (UCLA‐LS total score) were examined using Spearman correlations, computed separately per group. In an additional analysis in the PDD group, associations with early adversity (CTQ emotional neglect and emotional abuse) were examined. To control for multiple testing across these 24 main correlations, Benjamini–Hochberg false discovery rate (FDR) correction was applied, and FDR‐adjusted *p* values are reported. Supplementary analyses probed the robustness of the observed association patterns: differences between the groups' correlation coefficients were tested using Fisher's *r*‐to‐*z* tests, and, for all significant duration‐above‐threshold associations, partial Spearman correlations controlling for the person‐level mean of the same EMA item were computed. Additionally, we modeled the probability of leaving a high‐loneliness state at the next assessment using complementary log–log mixed models with participant random intercepts, group (PDD vs. HC), and controlling for person‐mean‐centered current loneliness. Hazard ratios <1 indicate lower exit probability in PDD. Given their supplementary and descriptive character, these analyses are reported uncorrected. Statistical significance was set at α = 0.05, and all tests were two‐tailed.

## 3 Results

### 3.1 Baseline Characteristics of the Sample

The sample consisted of 100 participants, comprising 58 inpatients with PDD (mean age 37.8 years, 50.0% female gender, and 15.8 mean education years) and 42 HC (mean age 38.8 years, 50.0% female gender, and 16.0 mean education years). As expected, trait loneliness (UCLA‐LS including all three subscales), early adversity (CTQ total score including emotional neglect and abuse scores), as well as depressive symptom severity (BDI‐II total score) were significantly higher in PDD than HC (Table 1).

**TABLE 1:** Demographic and clinical characteristics.

	PDD (*n* = 58)	HC (*n* = 42)	Cohen's *d*(95% CI)	*p* value
**Demographics**				
Age, mean (SD)	37.8 (13.1)	38.8 (15.3)	−0.07 (−0.50, 0.35)	0.737
Female, *n* (%)	29 (50.0%)	21 (50.0%)	φ = 0.00	1.000
Education years, mean (SD)	15.8 (4.1)	16.0 (2.9)	−0.05 (−0.46, 0.37)	0.814
**Trait loneliness**				
Total, mean (SD)	3.0 (0.7)	1.7 (0.6)	1.89 (1.40, 2.37)	< 0.001
Feelings of loneliness, mean (SD)	3.0 (0.8)	1.6 (0.7)	1.77 (1.30, 2.24)	< 0.001
Emotional isolation, mean (SD)	2.8 (1.0)	1.6 (0.7)	1.38 (0.93, 1.82)	< 0.001
Social isolation, mean (SD)	3.3 (0.6)	1.9 (0.6)	2.07 (1.57, 2.56)	< 0.001
**Clinical characteristics**				
BDI‐II, mean (SD)	32.7 (10.7)	3.3 (4.0)	3.43 (2.79, 4.05)	< 0.001
Age at depression onset, mean (SD)	14.9 (8.1)	—	—	—
Inpatient treatments, mean (SD)	3.1 (2.3)	—	—	—
CTQ total score, mean (SD)	52.0 (17.8)	36.4 (14.1)	0.95 (0.50, 1.39)	< 0.001
CTQ emotional abuse, mean (SD)	13.6 (6.2)	7.9 (3.9)	1.05 (0.61, 1.49)	< 0.001
CTQ emotional neglect, mean (SD)	15.1 (5.2)	9.6 (4.7)	1.10 (0.66, 1.54)	< 0.001

*Note*: Trait loneliness measured with UCLA loneliness scale.

Abbreviations: BDI‐II, Beck Depression Inventory second version; CTQ, Childhood Trauma Questionnaire; HCs, healthy controls; PDD, persistent depressive disorder.

Cohen's *d* (95% CI) for continuous variables, PDD minus HC. For sex, φ from chi‐squared. Independent‐samples *t*‐tests (Welch) for *p* values of continuous rows; chi‐squared for sex.

### 3.2 State Loneliness in Persistent Depressive Disorder and Healthy Controls

In line with our hypothesis, the PDD group showed significantly higher values on both state loneliness indices. This included higher mean state loneliness levels of 44.3 (SD = 17.4) compared to 10.9 (SD = 13.0) in HC (*p* < 0.001; *d* = 2.12, 95% CI [1.63, 2.62]) and longer durations of high‐loneliness states (*p* < 0.001; *d* = 0.84, 95% CI [0.42, 1.25]), lasting on average 3.5 (SD = 4.0) consecutive prompts in PDD versus 0.8 (SD = 1.6) consecutive prompts in HC. Figure 1 depicts state loneliness in PDD and HC. The same pattern held for state emotional and social isolation items (all *p* ≤ 0.004; visualization and effect sizes in Figures S1 and S2). Group differences in person‐mean values were of comparable magnitude in both assessment weeks, and within‐group changes between weeks were nonsignificant in PDD (Table S1).

**FIGURE 1:**
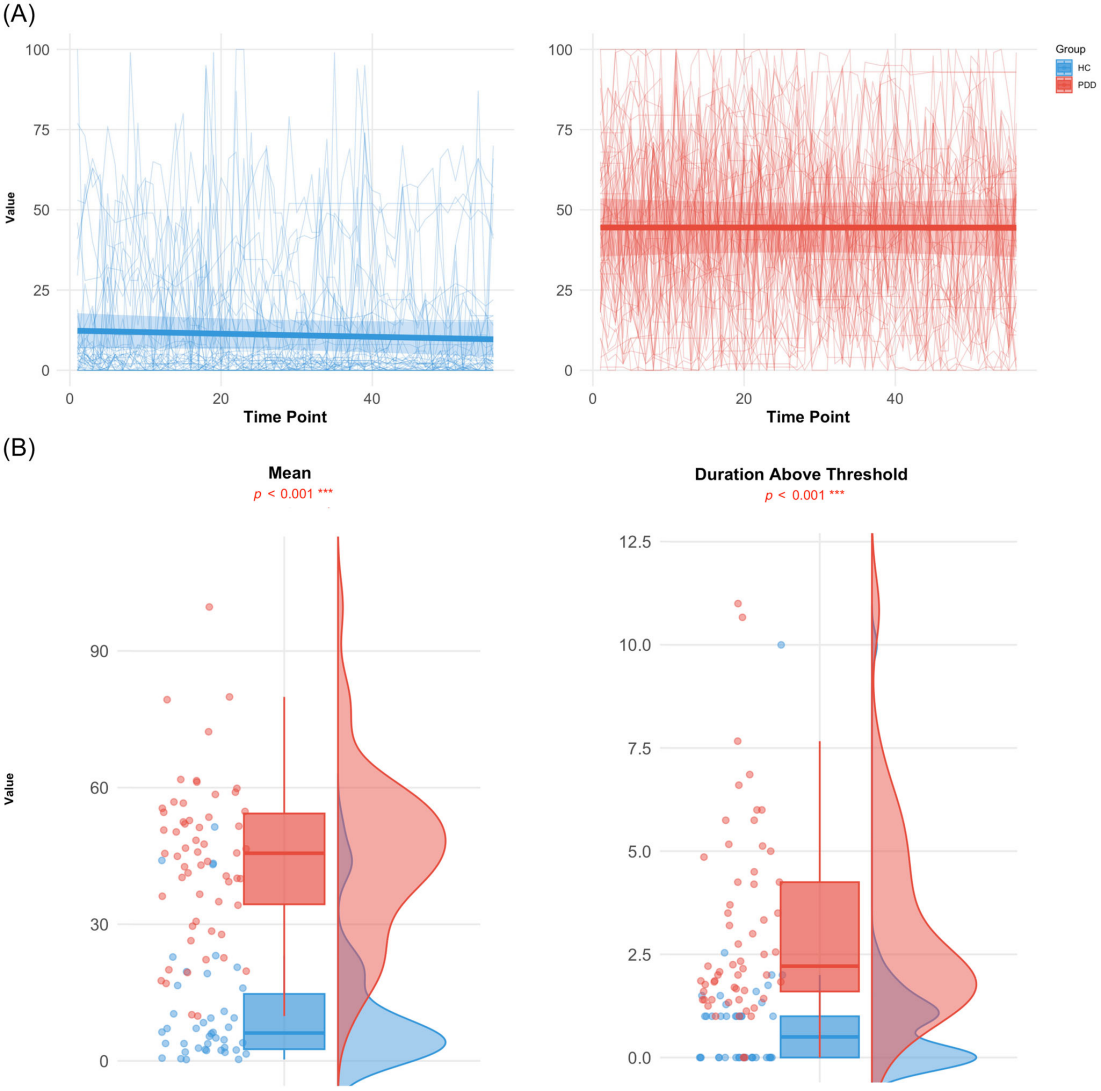
State loneliness in persistent depressive disorder and healthy controls. (A) Spaghetti plots of state loneliness measurements, thick lines indicate group‐level trends with error bands representing 95% confidence intervals. Group‐level trend lines were estimated using mixed‐effects models (state loneliness ∼ time × Group + (1 + time | participant ID)) and are shown for visualization only. (B) Raincloud plots depicting state loneliness (1) means and (2) duration above the 75th percentile of state loneliness values across all participants from both groups. Differences between groups were assessed via independent samples *t*‐tests (Cohen's *d*: mean = 2.12, 95% CI [1.63, 2.62]; duration above threshold = 0.84, 95% CI [0.42, 1.25]). *p < 0.05, **p < 0.01, ***p < 0.001.

Decomposing the duration index, 96.6% of PDD versus 50.0% of HC participants showed at least one above‐threshold state loneliness observation (*p* < 0.001, Fisher's exact test), with 6.4 (SD = 3.4) versus 1.6 (SD = 2.9) above‐threshold episodes per participant and run lengths of 3.7 (SD = 4.0) versus 1.7 (SD = 1.9) consecutive prompts among participants with at least one episode; these runs spanned on average 14.6 h (SD = 8.8) in PDD versus 7.7 h (SD = 10.0) in HC. The patterns were comparable for state emotional and social isolation (Table S2). In the supplementary discrete‐time hazard model, inpatients with PDD were less likely than HCs to leave high‐loneliness states at the next assessment (state loneliness: HR = 0.50 [0.28, 0.89], *p* = 0.018; state emotional isolation: HR = 0.53 [0.32, 0.87], *p* = 0.011; state social isolation: HR = 0.52 [0.31, 0.87], *p* = 0.014).

### 3.3 Associations of State Loneliness With Trait Loneliness and Early Adversity

Regarding trait loneliness (Table 2, Panel A), the mean level of emotional isolation states was significantly associated with trait loneliness in both groups (PDD: *r* = 0.65, *p*(FDR) < 0.001; HC: *r* = 0.54, *p*(FDR) = 0.003), as was the mean level of social isolation states (PDD: *r* = 0.42, *p*(FDR) = 0.015; HC: *r* = 0.41, *p*(FDR) = 0.035). The mean level of state loneliness was significantly associated with trait loneliness in HC (*r* = 0.47, *p*(FDR) = 0.015) but not in PDD (*r* = 0.21). The duration of high‐emotional isolation states was associated with trait loneliness in PDD (*r* = 0.60, *p*(FDR) < 0.001) but not in HC (*r* = 0.20). Exploratory comparisons of correlation coefficients between groups (Fisher's *z*; Table S3) yielded one nominally significant difference, for the duration of high‐emotional isolation states (*z* = 2.11, *p* = 0.035); no other contrast reached significance. Regarding our analysis on early adversity (Table 2, Panel B), higher mean levels (*r* = 0.40, *p*(FDR) = 0.015) and longer durations above threshold (*r* = 0.30, *p*(FDR) = 0.035) of emotional isolation states were associated with CTQ emotional neglect, but not with emotional abuse (*r* = 0.24, *r* = 0.16). Other state loneliness indices were not significantly associated with emotional neglect or abuse. For all significant duration above‐threshold associations, partial correlations controlling for mean levels were nonsignificant (all *p* ≥ 0.354; Table S4).

**TABLE 2:** Correlation coefficients of state loneliness with trait loneliness and early adversity.

Panel A: Trait loneliness correlations (UCLA‐LS total score)			
**EMA item**	**Index**	**PDD**	**HC**
State loneliness	Mean	0.21	0.47*
	Duration above threshold	0.09	0.17
State emotional isolation	Mean	0.65***	0.54**
	Duration above threshold	0.60***	0.20
State social isolation	Mean	0.42*	0.41*
	Duration above threshold	0.37*	0.09

Panel B: Early adversity correlations in the PDD group (CTQ emotional neglect and abuse scales)			
**EMA item**	**Index**	**Emotional neglect**	**Emotional abuse**
State loneliness	Mean	0.10	0.12
	Duration above threshold	0.03	0.12
State emotional isolation	Mean	0.40*	0.24
	Duration above threshold	0.30*	0.16
State social isolation	Mean	0.13	0.08
	Duration above threshold	0.22	0.07

*Note*: Spearman correlations. Mean = person‐level means of EMA state values; Duration above threshold = mean length of consecutive runs above the 75th percentile of EMA values pooled across all participants of both groups.

Abbreviations: CTQ, Childhood Trauma Questionnaire; EMA, ecological momentary assessment; HCs, healthy controls; PDD, persistent depressive disorder; UCLA‐LS, UCLA Loneliness Scale.

*p* values were FDR corrected. *p < 0.05, **p < 0.01, ***p < 0.001.

## 4 Discussion

To our knowledge, this is the first study to assess state loneliness in a clinical group of inpatients with PDD compared to a matched HC group. In line with our hypothesis, the PDD group showed elevated mean state loneliness and more persistent high‐loneliness states, whereas high‐loneliness states in HC were typically shorter. State emotional isolation emerged as the most consistent link between daily‐life experience and trait loneliness and was also associated with recalled emotional neglect in PDD.

The central finding concerns the elevation and temporal persistence of loneliness states in PDD. High‐loneliness states persisted across an average of 3.5 consecutive prompts, and among participants with at least one episode, spanned close to 15 h. Because mean level and duration above threshold were strongly coupled in our data, and duration associations did not remain significant when controlling for mean levels, we interpret the elevation and duration metrics as facets of a broader profile of persistent high‐loneliness states rather than as separable mechanisms. The discrete‐time exit model provides more direct evidence for temporal persistence, because it tests whether participants leave a high‐loneliness state at the next assessment while accounting for their current loneliness level. Together, these findings move beyond the well‐replicated observation that trait loneliness is elevated in PDD [7, 8, 23, 27] and describe how this elevation is organized in time. Thus, the findings suggest that state loneliness in PDD is characterized not only by greater severity, but also by a greater tendency to remain high over time.

Several mechanisms could contribute to this persistence. McCullough's model of PDD [28] proposes that negative interpersonal experiences impair the ability to (re)connect with others. This may help explain why state loneliness persists in PDD. However, individuals with PDD may also have smaller networks and lower relationship quality [4, 23], may feel misunderstood by others or expect not to receive support [31], and members of their social environment may themselves withdraw when confronted with persistent distress. The inpatient setting itself may further contribute: hospitalization restructures daily routines and separates patients from their usual social environment, which may affect both the level and the persistence of loneliness states. Conversely, the shorter high‐loneliness states in HC may simply reflect transient situational triggers, such as a farewell or lacking company at a specific moment, rather than superior regulation. This study cannot differentiate between these mechanisms.

The second main finding concerns state emotional isolation. Whereas the single state loneliness item (“At the moment I feel lonely.”) was only weakly and nonsignificantly related to trait loneliness in PDD, state emotional isolation, that is, the momentary perception of lacking people to turn to, was robustly associated with trait loneliness in both groups. Again, several post hoc explanations are possible. Emotional isolation may capture a more concrete perception of available social support, whereas directly identifying oneself as lonely may be influenced by stigma or differences in how the item is interpreted [1, 2]. Differences between a single EMA item and the 20‐item trait measure may also contribute. Because only one of six exploratory comparisons of correlations differed between groups, the group‐specific patterns should be interpreted cautiously.

Regarding early adversity, the most direct finding is an association between recalled emotional neglect and the mean level of state emotional isolation. The corresponding association with the duration of high‐emotional isolation states did not hold after adjustment for mean level, and no association emerged for emotional abuse. This refines the trait‐level literature: meta‐analytically, emotional neglect and emotional abuse were both strong correlates of loneliness [30], a pattern our group found across a population‐based and a clinical sample including PDD [23]. At the level of daily‐life experience, however, only emotional neglect was associated with state emotional isolation. Although this discrepancy should not be overinterpreted given the sample size, it is consistent with attachment‐based accounts in which the absence of emotional responsiveness shapes enduring expectations of interpersonal unavailability [29, 30], which may manifest in adulthood as the momentary perception of having no one to turn to.

Taken together, these findings are consistent with a potential developmental pathway of loneliness in PDD, as suggested by McCullough's model [28]: early emotional neglect may foster expectations of interpersonal unavailability that may manifest in daily life as elevated emotional isolation states, which in turn may be closely tied to trait loneliness. Nevertheless, this potential pathway remains hypothetical: early adversity was assessed retrospectively, and recalled adversity reflects the adult's current representation of childhood experiences rather than documented exposure [30]; moreover, we did not formally assess whether loneliness states mediate the association between early adversity and trait loneliness. Prospective studies are needed to test these relationships and their direction over time.

Clinically, the findings align with treatment models that target interpersonal avoidance and disconnection and are grounded in accounts of early adversity, most directly CBASP [7, 15, 28]. Notably, patients with early adversity have been found to benefit particularly from CBASP, also regarding loneliness outcomes [7, 39, 40]. EMA‐based monitoring of persistent high‐emotional isolation states could complement trait assessment for loneliness‐focused interventions [15, 26].

Several limitations should be acknowledged. First, the hypotheses and analyses were not preregistered, and this report is a secondary analysis of a larger study for which recruitment is ongoing; all findings should, therefore, be considered exploratory and hypothesis‐generating. Second, patients with PDD were assessed as inpatients, whereas HC lived in their usual environments; the hospital setting alters the social context and may have influenced state loneliness levels and their temporal persistence independently of the disorder. Third, state and trait loneliness were measured with different granularity, that is, single EMA items versus a validated 20‐item scale; moreover, the state emotional isolation item asks for “enough people” to turn to, adding a quantitative element to a construct for which a single confidant may suffice. Fourth, the indices depend on operationalization choices, such as the global 75th‐percentile threshold, and may vary under alternative operationalizations. Fifth, the moderate sample size limited statistical power, particularly for comparisons of correlation coefficients between groups. Finally, the two 1‐week EMA windows may have reduced sensitivity to slower temporal processes.

## 5 Conclusion

In this EMA study, loneliness in inpatients with PDD was elevated and occurred in persistent high‐loneliness states, whereas high‐loneliness states in HC were shorter. State emotional isolation (the momentary perception of lacking people to turn to) emerged as the most consistent link between daily‐life experience, trait loneliness, and recalled emotional neglect. These findings are consistent with the CBASP model, suggesting that early emotional adversity may contribute to emotional isolation states in daily life and trait loneliness in PDD. This potential pathway should be tested prospectively in preregistered studies with larger samples and longer observation windows, including whether strengthening perceived emotional connection can shorten high‐loneliness states in PDD.

## Author Contributions

**Johannes Wolf**: conceptualization, data curation, methodology, supervision, writing – original draft, writing – review and editing. **Katrin Wörz**: data curation, methodology, project administration, writing – original draft. **Amelie Cronau**: data curation, project administration, writing – review and editing. **Franziska Midderhoff**: data curation, project administration, writing – review and editing. **Julia I. Kunz**: writing – review and editing. **Alma‐Sophia Merscher**: writing – review and editing. **Claus Vögele**: writing – review and editing. **Andrea Jobst**: writing – review and editing. **Stephan Goerigk**: methodology, writing – review and editing. **Frank Padberg**: conceptualization, supervision, writing – review and editing. **Matthias A. Reinhard**: conceptualization, supervision, writing – review and editing.

## Conflicts of Interest

Frank Padberg received honoraria for workshops from Kirinus Munich, AWIP Ulm, and Lindauer Psychotherapy Weeks related to CBASP. He is a member of the European Scientific Advisory Board of Brainsway Inc., Jerusalem, Israel, and the International Scientific Advisory Board of Sooma, Helsinki, Finland. He has received speaker's honoraria from Mag&More GmbH, the neuroCare Group, Munich, Germany, and Brainsway Inc. His lab has received support with equipment from neuroConn GmbH, Ilmenau, Germany, Mag&More GmbH and Brainsway Inc. All other authors declare no conflicts of interest.

## Supporting information




**Supporting Information Material**: nyas70436‐sup‐0001‐SuppMat.pdf

## Data Availability

The data that support the findings of this ongoing study are not openly available due to reasons of participant privacy and are available from the corresponding author upon reasonable request.
