# Supplementary material for: Sustained High‐Loneliness States in Persistent Depressive Disorder: An Ecological Momentary Assessment Study
Source: Ann N Y Acad Sci. 2026 Oct 23;1564(1):e70436. doi: 10.1111/nyas.70436 (PMC13692695; doi:10.1111/nyas.70436)
Supplement: Supplementary file 1 [file NYAS-1564-0-s001.pdf]

Supplement

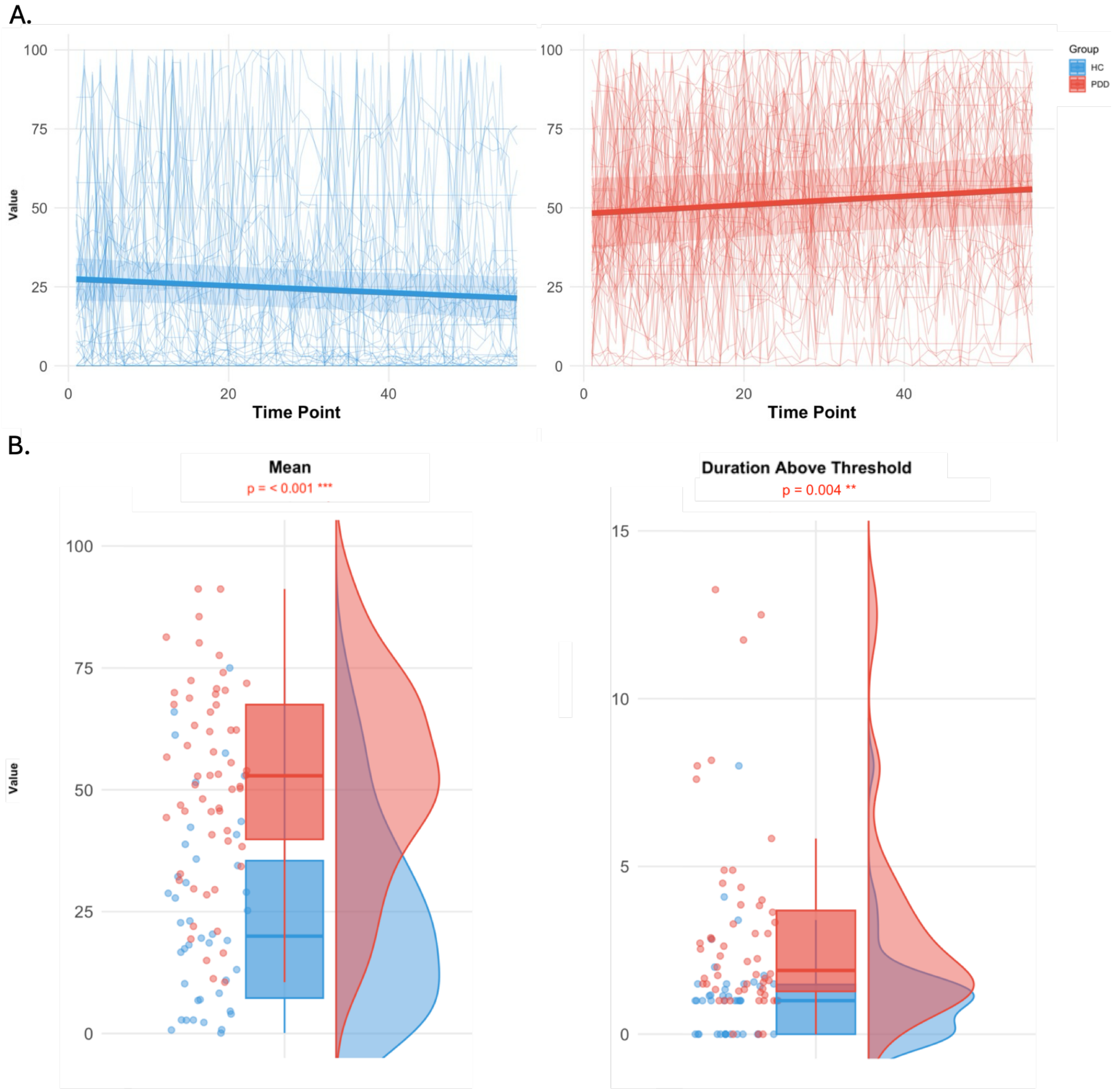

Supplementary Figure S1. State emotional isolation in persistent depressive disorder and healthy controls. A. Spaghetti plots of state **emotional isolation** measurements, thick lines indicate group-level trends. Group-level trend lines were estimated using mixed-effects models (state emotional isolation ~ time * Group + (1 + time | participant ID)) and are shown for visualization only. **B.** Raincloud plots depicting state **emotional isolation** (1) means and (2) duration above the 75th percentile of state emotional isolation values across all participants from both groups. Differences between groups were assessed via independent samples t-tests (Cohen's d: mean = 1.37, 95% CI [0.93, 1.82]; duration above threshold = 0.52, 95% CI [0.11, 0.92]).

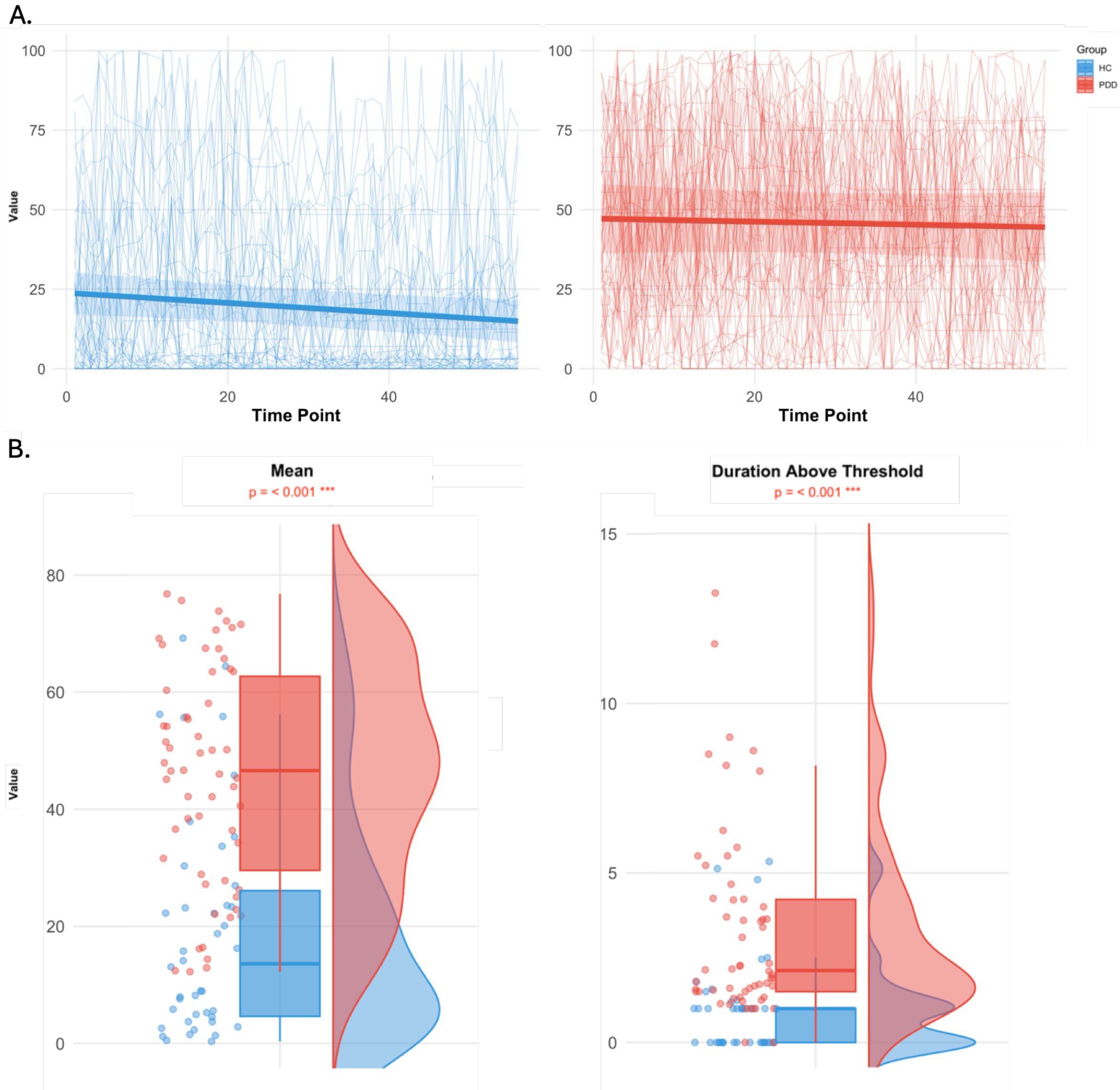

Supplementary Figure S2. State social isolation in persistent depressive disorder and healthy controls. A. Spaghetti plots of state **social isolation** measurements, thick lines indicate group-level trends. Group-level trend lines were estimated using mixed-effects models (state social isolation ~ time * Group + (1 + time | participant ID)) and are shown for visualization only. **B.** Raincloud plots depicting state **social isolation** (1) means and (2) duration above the 75th percentile of state social isolation values across all participants from both groups. Differences between groups were assessed via independent samples t-tests (Cohen's d: mean = 1.39, 95% CI [0.95, 1.84]; duration above threshold = 1.01, 95% CI [0.59, 1.43]).

Supplementary Table S1. State loneliness indices by assessment week

Panel A: Group comparison within each EMA week

EMA item	Week	PDD, M (SD)	HC, M (SD)	p
State loneliness	Week 1	44.6 (17.8)	11.7 (13.1)	< .001
	Week 5	43.0 (18.5)	10.4 (13.8)	< .001
State emotional isolation	Week 1	50.3 (19.4)	26.1 (20.3)	< .001
	Week 5	54.1 (22.1)	23.1 (20.8)	< .001
State social isolation	Week 1	46.7 (20.9)	22.0 (20.9)	< .001
	Week 5	43.9 (19.0)	16.9 (18.3)	< .001

Panel B: Within-group change from week 1 to week 5

EMA item	Group	Week 1, M (SD)	Week 5, M (SD)	p
State loneliness	PDD	44.6 (17.8)	43.0 (18.5)	.765
	HC	11.7 (13.1)	10.4 (13.8)	.169
State emotional isolation	PDD	50.3 (19.4)	54.1 (22.1)	.067
	HC	26.1 (20.3)	23.1 (20.8)	.065
State social isolation	PDD	46.7 (20.9)	43.9 (19.0)	.342
	HC	22.0 (20.9)	16.9 (18.3)	< .001

Note. Person-mean values of the respective EMA item, computed separately for each assessment week. Panel A: independent-samples t-tests. Panel B: paired t-tests. Uncorrected p-values. EMA = ecological momentary assessment; PDD = persistent depressive disorder; HC = healthy controls.

**Supplementary Table S2. Decomposition of duration above threshold**						
EMA item	Ever above, PDD, n (%)	Ever above, HC, n (%)	Number of episodes, PDD	Number of episodes, HC	Run length in prompts per episode, PDD, m (SD)	Run length in prompts per episode, HC, m (SD)
State loneliness	56 (96.6%)	21 (50.0%)	6.4 (3.4)	1.6 (2.9)	3.7 (4.0)	1.7 (1.9)
State emotional isolation	55 (94.8%)	27 (64.3%)	5.2 (3.6)	3.0 (3.9)	4.5 (8.1)	1.7 (1.5)
State social isolation	56 (96.6%)	23 (54.8%)	5.9 (3.1)	2.3 (3.4)	3.4 (2.8)	1.8 (1.4)

Note. Ever above = at least one observation > threshold. Run length per episode = mean run length among participants with ≥1 episode. Among participants with at least one above-threshold episode, runs spanned on average 14.6 hours (SD = 8.8) in PDD versus 7.7 hours (SD = 10.0) in HC for state loneliness, 15.2 hours (SD = 10.5) versus 8.6 hours (SD = 5.6) for state emotional isolation, and 17.7 hours (SD = 15.7) versus 8.4 hours (SD = 8.7) for state social isolation.

Supplementary Table S3. Group comparisons of correlation coefficients between PDD and HC

EMA item	Index	r PDD	r HC	z	p
State loneliness	Mean	.21	.47	-1.26	.207
	Duration above threshold	.09	.17	-0.36	.716
State emotional isolation	Mean	.65	.54	0.71	.477
	Duration above threshold	.60	.20	2.11	.035
State social isolation	Mean	.42	.41	0.05	.958
	Duration above threshold	.37	.09	1.27	.205

Note. Fisher's r-to-z tests comparing independent Spearman correlations of EMA state indices with the UCLA-LS total score between PDD (n = 58) and HC (n = 42), using the Fieller/Zar variance correction for rank correlations. Negative z values indicate a larger coefficient in HC. EMA = ecological momentary assessment; PDD = persistent depressive disorder; HC = healthy controls.

Supplementary Table S4. Partial Spearman correlations of duration above threshold, controlling for person-level mean levels

Panel A: Trait loneliness correlations

EMA item	Group	r duration (zero-order)	r mean–duration	Partial r	p
State loneliness	PDD	.09	.83	-.14	.354
	HC	.17	.64	-.15	.371
State emotional isolation	PDD	.60	.86	.12	.411
	HC	.20	.64	-.23	.165
State social isolation	PDD	.37	.84	.05	.749
	HC	.09	.68	-.27	.110

Panel B: Childhood emotional neglect correlation

EMA item	Group	r duration (zero-order)	r mean–duration	Partial r	p
State emotional isolation	PDD	.30	.86	-.09	.528

Note. Partial correlations control for the person-level mean of the same loneliness item. Duration associations largely overlap with elevated mean levels when mean and duration are strongly coupled, consistent with interpreting both indices as facets of a common state loneliness construct. EMA = ecological momentary assessment.
